# Detection of *Mycobacterium ulcerans* with IS*2404* loop-mediated isothermal amplification and a fluorescent reporter probe

**DOI:** 10.1128/aem.00270-25

**Published:** 2025-04-16

**Authors:** Jean Y. H. Lee, Jessica L. Porter, Maria Globan, Caroline J. Lavender, Yinhua Zhang, Nathan A. Tanner, Emma C. Hobbs, Andrew H. Buultjens, Timothy P. Stinear

**Affiliations:** 1 Department of Microbiology and Immunology, The University of Melbourne at the Doherty Institute for Infection and Immunity 198084, Parkville, Victoria, Australia; 2 Department of Infectious Diseases, Monash Health 2538, Clayton, Victoria, Australia; 3 WHO Collaborating Centre for Mycobacterium ulcerans, Mycobacterium Reference Laboratory, Victorian Infectious Diseases Reference Laboratory, Doherty Institute, Melbourne, Victoria, Australia; 4 Applied Molecular Biology Research Division, New England Biolabs, Ipswich, Massachusetts, USA; 5 Department of Veterinary Biosciences, Melbourne Veterinary School, Faculty of Science, University of Melbourne, Werribee, Victoria, Australia; 6 Department of Infectious Diseases, University of Melbourne, The University of Melbourne at the Doherty Institute for Infection and Immunity, Melbourne, Victoria, Australia; Royal Botanic Gardens, Surrey, United Kingdom

**Keywords:** *Mycobacterium ulcerans*, Buruli ulcer, LAMP, isothermal amplification, fluorescent probe, diagnostics, IS*2404*

## Abstract

An exquisitely sensitive quantitative PCR (qPCR) assay targeting the high-copy number insertion sequence, IS*2404*, is the gold standard diagnostic test for *Mycobacterium ulcerans*, the agent of the neglected tropical skin disease Buruli ulcer. Here, we designed and tested an alternative *M. ulcerans* diagnostic test, a fluorescent probe-based, loop-mediated isothermal amplification (P-LAMP) assay that also targets IS*2404*. Benchmarked against IS*2404* qPCR, P-LAMP was equally specific and nearly as sensitive (analytical sensitivity of four vs two *M. ulcerans* genome copies). Clinical and environmental specimen validation against IS*2404* qPCR showed P-LAMP had 100% sensitivity and specificity. P-LAMP was twice as fast as qPCR with an average time-to-positive at the limit-of-detection of 19 minutes. P-LAMP targeting IS*2404* is a versatile assay that addresses the performance issues of previously described IS*2404* LAMP formats. This study tackles a key research priority for Buruli ulcer and represents another avenue for the development of rapid and accessible molecular diagnostics for this neglected tropical disease.

**IMPORTANCE:**

Buruli ulcer is a neglected tropical disease caused by infection with *Mycobacterium ulcerans*. Correct diagnosis is essential before appropriate treatment for Buruli ulcer can be started. Development of a portable, easy-to-use diagnostic test for *M. ulcerans* has been identified by the World Health Organization as a research priority. Buruli ulcer most commonly occurs in remote, rural areas; therefore, an ideal test is one that can be used at (or near) the point of care (community health centres) without the need for specialized laboratories. Here, we describe a molecular test using loop-mediated isothermal amplification (LAMP) to detect DNA specific to *M. ulcerans* and show that this new test has equivalent performance to the gold standard *M. ulcerans* PCR test currently used worldwide. Our new test is rapid (30 minutes to run), simple to perform, and could be further developed into a robust, portable format to provide accessible and affordable *M. ulcerans* diagnostics anywhere.

## INTRODUCTION

Buruli ulcer (BU) is a neglected tropical skin disease caused by infection with *Mycobacterium ulcerans*. In the mid-1990s, scientists discovered *M. ulcerans* harbored a highly repetitive DNA fragment that was recognized as a potential target for a diagnostic PCR assay (1). Investigation identified this fragment was part of an insertion sequence that was named IS*2404* (2). Repeated more than 200 times within the genome, the element appears restricted to the *M. ulcerans* group (a single mycobacterial lineage evolved from a *Mycobacterium marinum* recent common ancestor and defined primarily by the presence of the pMUM mycolactone plasmid), providing a highly specific target (1–3). The gold standard molecular diagnostic assay for *M. ulcerans* targets IS*2404* to produce a 59 bp amplicon (4), and this qPCR is the mainstay of *M. ulcerans* clinical molecular diagnostics and environmental surveillance (4–9). Attempts to adapt this assay into a robust format suitable for use outside a molecular laboratory have had limited success although recent advances have included utilization of portable, compact, battery-operated equipment and simplified DNA extraction and purification protocols (10, 11).

A rapid, relatively simple test for the diagnosis of BU that could be deployed in the field is one of the research priorities identified in the World Health Organization (WHO) *Road Map for Neglected Tropical Diseases 2021–2030* (12). Such a test would be used in active case finding activities and to inform prompt treatment of BU with appropriate antibiotics. In 2022, the WHO convened an expert panel to design a target product profile (TPP) for a rapid BU test suitable for use at the primary health care level (13). Some of the key minimum features outlined in the TPP included (i) suitable for use by nurse or lab technician; (ii) ≥90% sensitivity and specificity compared with IS*2404* qPCR; (iii) able to detect African *M. ulcerans* strains; (iv) suitable for swabs and fine-needle aspirate specimens; (v) <5 steps for sample preparation; (vi) same-day result; (vii) simple result interpretation; (viii) integrated internal control; (ix) simple detection instrumentation; (x) no reagent cold chain needed; (xi) cost-per-test <$1USD (13).

The loop-mediated isothermal amplification (LAMP) assay is easy to perform, sensitive, rapid, and conducted at a constant temperature (mitigating the need for thermocycling equipment), making it amenable to point-of-care testing (14–16). For these reasons, several research groups have explored the potential of LAMP for the detection of *M. ulcerans*, with variable success (17–21). Typically, a trade-off between specificity and sensitivity exists with conventional LAMP methods, as measures employed to increase sensitivity also introduce false positive results (16, 22). Here, we have designed and tested a LAMP assay that uses a TaqMan-style reporter probe with inclusion of locked nucleic acids (LNA) to improve stability through adjustment of probe melting temperatures (23), but without the requirement for enzymatic hydrolysis of nucleotides linked to the probe reporter fluorophore. This assay format overcomes some of the major limitations of LAMP and is an advance over previous LAMP assays described for BU diagnosis. These advantages include maintaining a single, closed tube format to prevent amplicon contamination and high assay specificity to preclude reporting false positive results. Probe-based LAMP also has the potential for single-tube multiplexing, whereby an internal positive control using a different fluorescent reporter probe is incorporated into every reaction to guard against false negative results.

A potential limitation of using specific reporter probes for LAMP assays is that they can exhibit reduced sensitivity compared to other LAMP amplicon detection systems. However, here we show that *M. ulcerans* LAMP assay performance is not compromised by configuring the assay to incorporate the probe format. Comparison with IS*2404* qPCR demonstrated a functionally equivalent analytical limit-of-detection and equivalent sensitivity and specificity for the clinical detection of *M. ulcerans*.

## MATERIALS AND METHODS

### Bacterial isolates

Bacterial strains used in this study and their associated references are listed in Table S1.

### Possum excreta samples

Structured possum excreta surveillance studies covering the Mornington Peninsula (70 km south of Melbourne), Geelong, and inner metropolitan Melbourne, Victoria, Australia, were conducted according to the standardized method described by Vandelanoote et al. (9), between 2022 and 2023. Voided possum excreta specimens were transported to the laboratory on cold packs within 24 hours of collection and then stored at −20°C until the time of DNA extraction. Based on previous surveillance results, 78 excreta were collected from sites at which possums with a high probability of *M. ulcerans* infection were known to reside. Genomic DNA from possum excreta was extracted using either the PowerSoil Pro kit (Qiagen Cat 47016) or a SPRI-bead based extraction method developed for the testing of possum excreta for *M. ulcerans* (11). DNA was stored at −20°C.

### Possum swab samples

Active trap-and-release and passive necropsy-based surveillance studies of wild native possums were conducted around Melbourne and Geelong between November 2021 and December 2022 (24). During these studies, dry flocked swabs (Copan 8155C1S) were used to sample cutaneous lesions and body cavities (oral, cloaca, pouch). Collected swabs (*N* = 16) were stored in individual tubes at −20°C until the time of DNA extraction. For extraction of genomic DNA, swabs were inoculated into 1 mL of phosphate-buffered saline and vortexed for 1 minute, and a 200 µL aliquot of the solution was then processed using a SPRI-bead based extraction method (11). Genomic DNA was stored at −20°C.

### Human clinical samples

Human clinical samples (*N* = 102) were submitted to the Victorian Infectious Diseases Reference Laboratory (VIDRL), Australia, collected between 2021 and 2024. Specimens were processed routinely at the time of submission, using the DNA extraction method and IS*2404* TaqMan qPCR previously described by Fyfe et al. (4) to test for the presence of *M. ulcerans*. Following testing by VIDRL, the remainder of the DNA extractions were stored at −20°C until utilized for this study.

### Genomic DNA extractions from pure bacterial cultures

Extraction of genomic DNA from mycobacterial species was performed as previously described (11). *Nocardia* and *Streptomyces* DNA extractions followed a specialized protocol for these species (25). For all other bacteria, the DNeasy Blood & Tissue kit (Qiagen Cat 69504) was used, following the appropriate protocol for Gram-positive (*Enterococcus faecium*, *Listeria monocytogenes,* and Staphylococci) or Gram-negative (*Dendrosporobacter quercicolus, Escherichia coli*, *Klebsiella pneumoniae,* and *Rouxiella chamberiensis*) bacteria. For *Staphylococcus aureus* (26) and *Staphylococcus epidermidis* (27), species-specific pretreatments with lysostaphin were included prior to using the DNeasy Blood & Tissue kit for DNA extraction. Genomic DNA extractions from *M. ulcerans* clinical isolate JKD8049 (3) were used as the positive control material for all experiments.

### IS*2404* LAMP plus locked nucleic acid probe (P-LAMP)

The oligonucleotide primers for IS*2404* LAMP were designed using LAMP Designer (www.optigene.co.uk) and the primer design function in Geneious Prime (v2024.0.7 www.geneious.com). A fluorescent reporter probe for the LAMP primer set was designed to bind the interior amplicon region of the LAMP amplicon using design rules as previously described (23). LNA bases were used in probe design to increase melting temperature and adjust for AT-rich regions (23).

Each 25 µL LAMP reaction consisted of 2× WarmStart Multi-Purpose LAMP/RT-LAMP 2× Master Mix (NEB M1700S), 40 mM guanidine hydrochloride (pH 8.0) (Sigma-Aldrich G3272-500G), 6 LAMP primers at standard concentrations (1.6 µM BIP, 1.6 µM FIP, 0.2 µM B3, 0.2 µM F3, 0.4 µM LoopB, and 0.4 µM of LoopF), 0.25 µM IS*2404* LAMP LNA probe, 5.0 µL sample DNA, and nuclease-free water to a total volume of 25 µL. No-template and *M. ulcerans* positive controls were included in every qPCR run. Sequences for the six IS*2404* LAMP primers and the LAMP LNA probe are shown in Table 1. Oligonucleotides were purchased from Integrated DNA technologies. LAMP reactions were run on the Mic qPCR Cycler (Bio Molecular Systems), implementing a 65°C incubation for 30 minutes with 30 second interval data collection at an emission wavelength of 540 nm (FAM channel). Run analyses were performed using Mic PCR software (v2.10.5 Bio Molecular Systems).

**TABLE 1:** Primer and probe sequences^*a*^

Primer/probe	Sequence (5′ to 3′)	Reference
IS*2404* qPCR		
IS*2404* TF	AAAGCACCACGCAGCATCT	(4)
IS*2404* TR	AGCGACCCCAGTGGATTG	(4)
IS*2404* TP	/6 FAM/CGTCCAACGCGATC/MGBNFQ/	(4)
IS*2404* P-LAMP		
BIP(B1c + B2)	TGGTCACTGTGGATGCGAT-GCATCAGGTAGTGCGACTTC	(28)
FIP (F1c + F2)	GGCACGTACGCAGGGAAT-GATTGGTGCTCGGTCAAC	(28)
B3	TTGGCTTGGTTGGACTTG	(28)
F3	CATCTCGTGTCGGTGTTC	(28)
LoopB	TCACCGCGAAGTTGATCTG	(28)
LoopF	CATTGCTTTTCTCGGCGAC	(28)
LNA probe	/6 FAM/TGG C+TG GTC +A+CT G+TG GA/3IABkFQ/	This study

^*a*^ Primers have been named according to the publications in which they were originally described, in which the IS*2404* TaqMan qPCR forward and reverse primers were named their reverse relative to the insertion sequence transposase gene orientation (4). The forward and reverse primers for the IS*2404* P-LAMP were also named the reverse of convention (28). “+” after a base indicates a locked nucleic acid (LNA)-modified base.

### IS*2404* TaqMan quantitative PCR

Primers were purchased from Integrated DNA technologies. Adapted from the method previously described (4), using primers targeting the IS*2404* insertion sequence in *M. ulcerans* (Table 1) with TaqMan Exogenous Internal Positive Control (IPC) Reagents—VIC Probe (Applied Biosystems 4308323) and SensiFAST probe No-ROX Kit (Bioline BIO-86005), each 20 µL reaction consisted of 2× SensiFAST Probe No-ROX mix, 0.4 µM IS*2404* TF, 0.4 µM IS*2404* TR, 0.1 µM IS*2404* TP, 10× TaqMan Exo IPC Mix, 50× TaqMan Exo IPC DNA, 3.2 µL of nuclease-free water, and 2 µL of sample DNA. No-template controls, *M. ulcerans* genomic DNA positive control, and TaqMan IPC block (indicates if PCR inhibition has occurred) were included in every qPCR run. Amplification and detection were performed using the following program: 95°C for 5 minutes, then 45 cycles of 95°C for 10 seconds, and 60°C for 20 seconds. A cycle threshold (Ct) ≤40 was interpreted as positive for the presence of *M. ulcerans*. This cut-off was in accordance with previous performance evaluations of the assay (9, 29). All IS*2404* qPCR testing was performed on the Mic PCR platform (Bio Molecular Systems), except the testing of DNA extractions from possum excreta and swabs collected from possums. For both these specimen types, the IS*2404* qPCR was performed on the QuantStudio 1 (Thermo Fisher Scientific) platform. Run analyses were performed using Mic PCR software (v2.10.5 Bio Molecular Systems) or Design & Analysis Software (v2.5.0 Thermo Fischer Scientific).

### Statistical analyses

Data comparisons and statistical tests were performed using GraphPad Prism (v10.2.0, www.graphpad.com).

## RESULTS

### IS*2404* LAMP with locked nucleic acid probe (P-LAMP) assay design and *in silico* analysis

A conventional LAMP assay using six primers was designed to target a 173 bp fragment of IS*2404* (nucleotide positions 634–806 of the 1,366 bp element). The location of the four amplifying oligonucleotides and the two strand-displacing oligonucleotides relative to the gold standard IS*2404* TaqMan primer and probe binding sites are shown in Fig. S1. To check primer specificity within *M. ulcerans*, *in-silico* scanning of the complete chromosomes of *M. ulcerans* strains JKD8049 (Genbank accession: CP085200, Australian isolate) and Agy99 (Genbank accession: CP000325, African isolate) were performed. These analyses revealed 204 hits for JKD8049 and 206 hits for Agy99, consistent with the known chromosomal copy numbers of IS*2404* for each strain (3) and indicated suitable specificity of the primers. The designed probe hybridized within the interior amplicon region between F1 and B1c, overlapping the 5′ end of B1c (Fig. S1), and had four LNA bases (Table 1). After initial performance tests demonstrated good signal strength and no inhibition of the LAMP reaction (data not shown), we proceeded with assay validation.

### The IS*2404* P-LAMP has reproducible performance and a limit-of-detection comparable to IS*2404* qPCR

**Fig 1:**
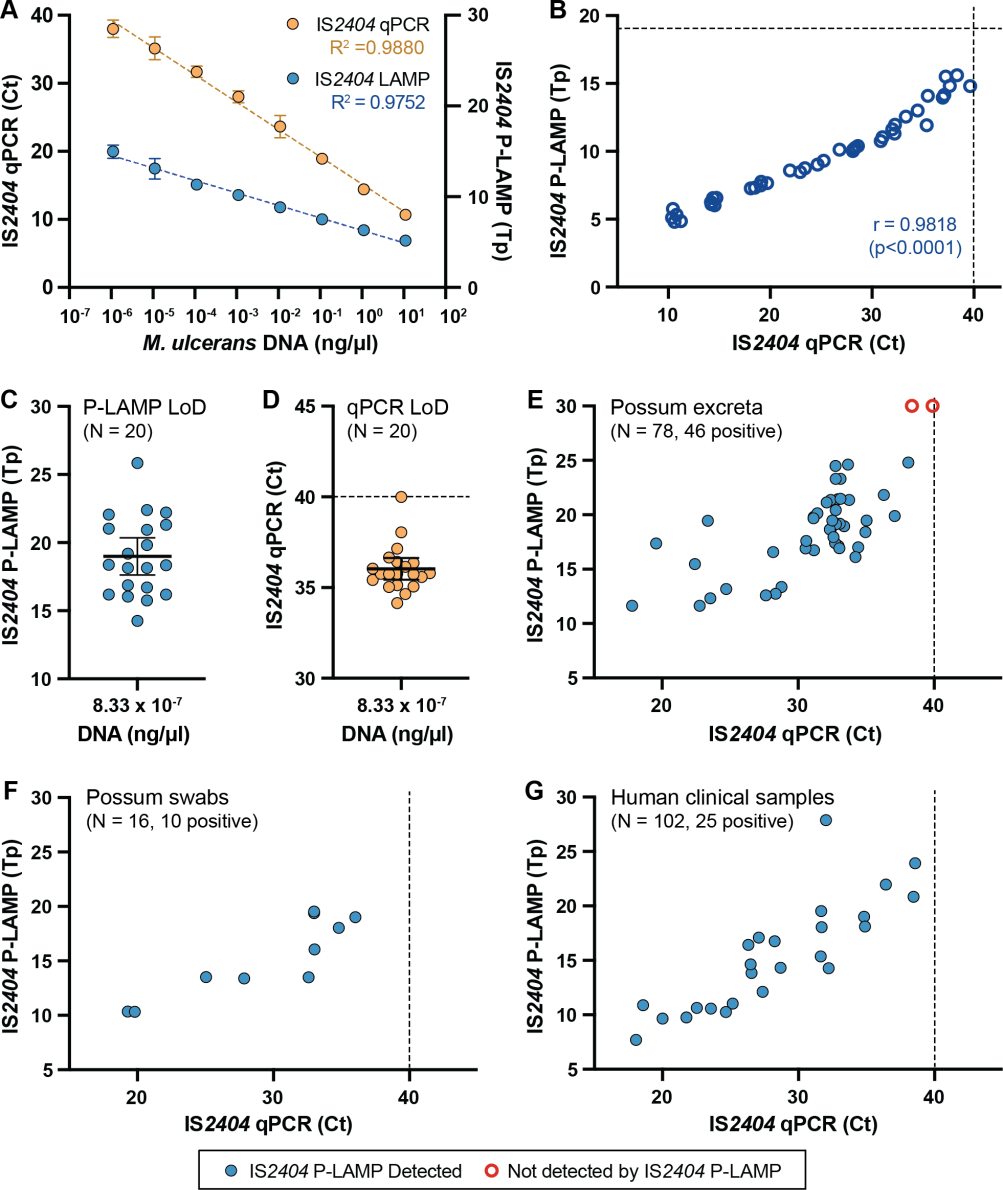
Validation of the IS*2404* probe LAMP (P-LAMP) for detection of *M. ulcerans*. (A) Ten-fold dilution series of *M. ulcerans* DNA (logarithmic concentration plotted) when assayed by both IS*2404* qPCR (yellow circles) and IS*2404* P-LAMP (blue circles). Dilution series performed in quintuplet. Plotted circles show the mean value of replicates at the dilution tested, expressed in cycle threshold (Ct) for IS*2404* qPCR and time-to-positive (Tp; plotted in decimal minutes) for IS*2404* P-LAMP. Error bars indicate the 95% confidence interval. Dotted yellow and blue lines indicate the regression lines for the IS*2404* qPCR and P-LAMP, respectively.** (B)** The IS*2404* qPCR Ct value of each dilution series replicate plotted against its corresponding Tp value when the same replicate was assayed with IS*2404* P-LAMP. Pearson correlation coefficient *r* = 0.9818. The null hypothesis (no correlation between the two assays) was rejected for *P* < 0.01 (Pearson correlation coefficient, two-tailed *P*-value) ****P < 0.0001. A vertical dotted line indicates the Ct ≤ 40 cut-off for the IS*2404* qPCR, above which samples are interpreted as negative. A horizontal dotted line shows the IS*2404* P-LAMP limit-of-detection (LoD). (**C)** LoD of the IS*2404* P-LAMP determined by the lowest dilution at which 20 of 20 replicates returned a positive result. Blue circles show each replicate detected. The bold horizontal bar indicates the mean, and error bars indicate the 95% confidence interval. (**D)** LoD of the IS*2404* qPCR. Yellow circles represent each replicate detected. A bold horizontal bar indicates the mean, and error bars indicate the 95% confidence interval. The horizontal dotted line indicates the Ct ≤ 40 cut-off for the IS*2404* qPCR. (**E)** Performance of IS*2404* P-LAMP compared to qPCR to detect *M. ulcerans* in genomic DNA extractions from native Australian possum excreta specimens. The vertical dotted line indicates the Ct ≤ 40 cut-off for the IS*2404* qPCR. The 32 samples that tested negative by both methods are not shown. Red circles indicate P-LAMP false negatives. (**F)** Performance of IS*2404* P-LAMP compared to qPCR to detect *M. ulcerans* in DNA extractions from swabs collected from possums. The six samples that tested negative by both assays are not shown. (**G)** Performance of IS*2404* P-LAMP compared to qPCR to detect *M. ulcerans* in DNA extractions from human clinical specimens. The 77 samples that tested negative by both methods are not shown.

To assess the performance of the IS*2404* P-LAMP, it was compared to the gold standard IS*2404* Taqman qPCR by assaying quintuplet log_10_ dilution series from 10^−1^ to 10^−10^ of *M. ulceran*s genomic DNA (Fig. 1A). P-LAMP performance was very similar to qPCR, both assays detected five of five samples up to the 10^−8^ dilution (1.11 × 10^−6^ ng/µL), and none of the 10^−9^ or 10^−10^ dilutions. Despite LAMP being regarded as a qualitative rather than a strictly quantitative assay, there was significant correlation between the time-to-positive (Tp) values for IS*2404* P-LAMP and the cycle threshold (Ct) values for IS*2404* qPCR (Pearson correlation coefficient, *r* = 0.9818; two-tailed *P*-value, *P* < 0.0001; Fig. 1B).

To establish the limit-of-detection (LoD) of the IS*2404* P-LAMP compared to IS*2404* qPCR, we tested the lowest concentration of *M. ulcerans* genomic DNA at which 20 of 20 replicates were reported positive result for each method. Two further dilutions (¾ and ½) of the 10^−8^ DNA dilution were tested, corresponding with 8.33 × 10^−7^ ng/µL and 5.55 × 10^−7^ ng/µL of *M. ulcerans* genomic DNA, respectively (Fig. S2). At a concentration of 8.33 × 10^−7^ ng/µL, both assays successfully detected 20/20 replicates. For IS*2404* P-LAMP, the mean Tp was 18.99 decimal minutes (95% CI 17.63–20.35) (Fig. 1C; Fig. S2A). For the IS*2404* qPCR, the mean Ct of the 20 replicates was 36.03 (95% CI 35.43–36.63) (Fig. 1D; Fig. S1B). Of note, one replicate had a Ct value of 39.99, just below the assay cut-off of Ct ≤ 40, above which results are considered “not detected.”

When the concentration of *M. ulcerans* genomic DNA was diluted to 5.55 × 10^−7^ ng/µL, both assays were less reliable. The IS*2404* P-LAMP detected 17/20 replicates with a mean Tp of 18.33 decimal minutes (95% CI 17.63–20.35) (Fig. S2A). The IS*2404* qPCR detected 19/20 replicates with a mean Ct of 37.50 (95% CI 36.54–38.46). The single “not detected” replicate had a Ct of 44.16 (Fig. S2B). Based on these results, the LoD of the IS*2404* P-LAMP was determined to be 8.33 × 10^−7^ ng/µL of *M. ulcerans* genomic DNA, comparable to the gold-standard IS*2404* qPCR. This concentration of DNA represents approximately four genome copies for P-LAMP vs two genome copies for qPCR based on an estimated weight of 6.25 fg for the *M. ulcerans* JKD8049 genome, and input volumes of 5 µL and 2 µL of template DNA for the IS*2404* P-LAMP and IS*2404* qPCR, respectively (29).

### IS*2404* P-LAMP concordance with IS*2404* qPCR when testing environmental samples

To validate the IS*2404* P-LAMP, we then tested genomic DNA extractions of relevant samples. Three specimen types were tested: possum excreta and swabs sampled from possums, both sourced from previous environmental surveillance studies, and human clinical samples. To allow direct comparison of performance, DNA extracted from all specimens was concurrently tested by the gold-standard IS*2404* qPCR.

A recognized major wildlife reservoir of *M. ulcerans*, native Australian possums shed *M. ulcerans* in their excreta, and they are also susceptible to clinical disease (30, 31). Seventy-eight possum excreta samples collected from sites at which possums were known to have a high probability of harbouring *M. ulcerans* were used in this study. Testing of the genomic DNA extractions from these samples with IS*2404* qPCR in an earlier study had identified 46 positives and 32 negatives (11) (Table S2).

The IS*2404* P-LAMP detected 44 of the 46 IS*2404* qPCR positive samples (Fig. 1E; Table S2). The two false negative samples by IS*2404* P-LAMP corresponded with IS*2404* qPCR Ct values close to the qPCR limit-of-detection (39.89 and 38.37). All 32 *M*. *ulcerans* IS*2404* qPCR negative samples were correctly identified by P-LAMP. Based on the above, IS*2404* P-LAMP was calculated to have a sensitivity of 95.83%, specificity of 100%, positive predictive value of 100%, and negative predictive value of 93.75% for testing genomic DNA extracted from possum excreta.

Sixteen swabs were collected from 10 possums, some with lesions suspicious of *M. ulcerans* infection (Table S3). Ten of the 16 swabs were IS*2404* qPCR positive, and the remaining 6 were negative. When the same DNA extractions were tested using the IS*2404* P-LAMP, there was 100% concordance for all 16 samples (Fig. 1F; Table S3).

### IS*2404* P-LAMP has 100% concordance with IS*2404* qPCR for human clinical specimens

To validate IS*2404* P-LAMP with human clinical samples, we tested stored genomic DNA that had been extracted from swab specimens collected from patients with suspected Buruli ulcers, submitted to the state reference laboratory between 2021 and 2024. A total of 102 genomic DNA extractions from human clinical samples were available. Twenty-five samples were IS*2404* qPCR positive, and 77 samples were IS*2404* qPCR negative (Table S4). Testing with IS*2404* P-LAMP showed 100% concordance for all specimens, representing 100% sensitivity and specificity, and 100% negative and positive predictive values.

### The IS*2404* P-LAMP detects *M. ulcerans* isolates originating from different countries

To confirm results of earlier *in silico* analyses and demonstrate the utility of the P-LAMP assay, we also tested genomic DNA from *M. ulcerans* isolates originating outside Australia (see Table S1). Strains included two clinical isolates from Ghana, Africa (32, 33), and one from Suriname, South America (34); and two fish-associated isolates, one from Belgium (35), and the other from the United States of America (36). All five isolates tested positive with P-LAMP.

### The IS*2404* LAMP probe is specific for the detection of *M. ulcerans*

To assess the specificity of IS*2404* P-LAMP, genomic DNA from a diverse range of mycobacteria and other bacteria were tested (Table 2). IS*2404* P-LAMP did not produce a positive result for any of the other 23 bacterial species tested, supporting the high specificity of this assay for *M. ulcerans*.

**TABLE 2:** Specificity testing of IS*2404* P-LAMP

Organism (strain)	IS*2404* P-LAMP	Strain reference
*Mycobacterium ulcerans* (JKD8049)	*+*	(3)
*Mycobacterium abscessus* (TPS8830)	ND^*b*^	Not published
*Mycobacterium bovis* (Danish 1331)	ND	(37, 38)
*Mycobacterium chimera* (DMG160013)	ND	(39)
*Mycobacterium fortuitum^a^*	ND	(2)
*Mycobacterium marinum* ("M” strain)	ND	(40)
*Mycobacterium smegmatis* (MC2155)	ND	(41, 42)
*Mycobacterium spongiae* (FSD4b-SM)	ND	(43)
*Mycobacterium terrae* (NCTC 10856/ATCC 15981)	ND	(44)
*Mycobacterium virginiense* (TPS8833)	ND	Not published
*Dendrosporobacter quercicolus* (DSM 1736)	ND	(45, 46)
*Enterococcus faecium* (Ef_AUS0233)	ND	(47)
*Escherichia coli* (Ec_SeRP62aI)	ND	(48)
*Klebsiella pneumoniae* (BPH05002)	ND	Not published
*Listeria monocytogenes* (EGDe)	ND	(49, 50)
*Nocardia brasiliensis* (AUSMDU00075719)	ND	Not published
*Nocardia testacea* (AUSMDU00041268)	ND	Not published
*Rouxiella chamberiensis* (DSM 28324)	ND	(51, 52)
*Staphylococcus aureus* (JE2)	ND	(53, 54)
*Staphylococcus aureus* (MW2)	ND	(55, 56)
*Staphylococcus epidermidis* (BPH0662)	ND	(27)
*Staphylococcus epidermidis* (BPH0736)	ND	(57)
*Streptomyces cacaoi* (AUSMDU00077794)	ND	Not published
*Streptomyces* spp. (AUSMDU00077800)	ND	Not published

^*a*^ Reference strain from the culture collection of the Queensland Mycobacterium Laboratory.

^*b*^ ND, not detected.

## DISCUSSION

In this study, we have shown that a TaqMan-style reporter probe in a *M. ulcerans* IS*2404* LAMP assay (P-LAMP) matches the performance of the gold standard IS*2404* qPCR. Clinical validation showed 100% specificity and sensitivity for P-LAMP (Fig. 1G). In a head-to-head comparison, the LoD for P-LAMP was very low, only slightly higher than qPCR [four *M. ulcerans* genome copies for P-LAMP (Fig. 1C) compared with two genome copies for qPCR (Fig. 1D; Fig. S2)]. Assay validation using environmental specimens showed 100% specificity and sensitivity ranging from 96% to 100% depending on specimen type (Fig. 1E and F). These results align with previous reports using IS*2404* LAMP assays for *M. ulcerans* diagnosis, where sensitivity and specificity (various calculation methods were used, so assay performance is not strictly comparable) ranged from 74% to 96% (17–21). The only two false negative results out of the 196 combined clinical and environmental samples tested with the P-LAMP assay occurred when testing genomic DNA extracted from possum excreta (Fig. 1E), such specimens are prone to carry over contamination with humic substances that inhibit molecular assays (11, 58). Furthermore, testing of these two samples identified them as being close to the limit-of-detection of the IS*2404* qPCR and, therefore, also close to the P-LAMP limit-of-detection (Fig. 1E). Despite being a qualitative rather than quantitative assay, there was a significant correlation between IS*2404* P-LAMP Tp and qPCR Ct values (Fig. 1B), pointing to the potential of P-LAMP outputs to provide an indication of bacterial burden in a specimen (Fig. 1E through G).

In keeping with the shorter assay runtime of LAMP compared to PCR, when run on the Mic PCR magnetic induction cycler, the IS*2404* P-LAMP had a runtime of 32 minutes 25 seconds, while the IS*2404* qPCR ran 61 minutes and 13 seconds. The Mic PCR platform was the preferred instrument to run the P-LAMP due to it suitability for in-field use, weighing only 2 kg, with wireless connectivity to a controlling laptop, and multiplexing ability with sensitive two or four channel optics. The compact nature of the device consumables (Fig. S3) required careful handling during test set-up to avoid contamination. Therefore, like diagnostic practice with the IS*2404* qPCR, testing of samples in duplicate is recommended.

For specificity testing, we selected nine other *Mycobacterium* species for IS*2404* P-LAMP to assess any cross reactivity with the primers-probe set due to genetic similarity. Four other bacterial species were also included for their potential to be found in environmental specimens like possum excreta. These bacteria included *Nocardia* and *Streptomyces* species that are prevalent in soil (59, 60), *D. quercicolus* (*Clostridium quercicolum* reclassified) (45) that was originally isolated from discoloured tissues in living oaks (46), and a *R. chamberiensis* isolate. Although *R. chamberiensis*, the first species in the taxon, was isolated as a contaminant in parenteral nutrition bags (51), the other three species in the genus described to date, *Rouxiella aceris*, *Rouxiella badensis*, and *Rouxiella sylvae*, have all been isolated from environmental settings including tree sap, blueberries, peat bog soil, and a swampy meadow that suggest a close association with soils and plant ecosystems (52, 61, 62). The remaining bacteria selected were common clinical pathogens. Our IS*2404* P-LAMP assay did not cross-react with any of the 23 tested species (Table 2). Of note, there was no cross reactivity with *M. marinum*, with which *M. ulcerans* shares > 98% nucleotide sequence identity and from which genomic population analyses show *M. ulcerans* evolved (33).

Although simple to perform, an inherent limitation of standard LAMP assays is methodology dependent upon the detection of accumulating DNA products using DNA intercalating or pH-sensitive dyes, or turbidity change; methods that can give false positive results and present constraints on sample type (23). The risk of false positives is particularly high when run-times are extended to boost assay sensitivity, which can result in non-specific DNA amplification due to interaction between DNA polymerase and primers in the absence of target nucleic acids (16, 22). Nucleic acid probes that hybridize to a specific region of the LAMP amplicon and fluoresce upon hybridization overcome this issue (23, 63), as demonstrated by the high specificity of the P-LAMP assay. A potential trade-off of probe-LAMP specificity can be the emission of a fluorescence signal that is orders of magnitude lower than a traditional LAMP intercalating dye reaction, resulting in loss of sensitivity (23, 63). However, as demonstrated by the relatively comparable analytical detection sensitivity of the IS*2404* P-LAMP with the gold-standard IS*2404* qPCR, this was not a concern with our assay.

A further advantage of probe-based LAMP is the potential for multiplexed reactions in a single tube by combining LAMP assays with different targets matched to fluorescent reporters with diverging emission spectra (63). The clinical robustness of our IS*2404* P-LAMP assay could be enhanced with the addition of a human DNA target to ensure that adequate sampling of lesions was performed, which would be particularly relevant for minimally invasive sampling with swabs or fine needle aspirate as specified by the WHO TPP for BU (13). Multiplexing with an internal positive control is another consideration. This would incorporate an unrelated DNA (or RNA) template into every IS*2404* P-LAMP test, which serves to identify false negative results due to assay inhibition or reagent failure. Both these assays have potential for further development.

Here, we demonstrate that an IS*2404* probe-based LAMP combines all the benefits of conventional LAMP, such as speed and isothermal amplification, with the probe associated benefits of specificity and potential for multiplexing. The IS*2404* P-LAMP assay is a solid foundation for further development to achieve the minimum criteria outlined by the WHO TPP for a rapid diagnostic test for BU. In its current format, this qualitative assay that detects *M. ulcerans* DNA can be conducted by a laboratory technician, performs above the stated ideal of >90% sensitivity and >90% specificity compared to qPCR, has demonstrated strain specificity for both the minimum criteria of African isolates and ideal criteria of international strains, can process both swabs (advanced stage ulcerated lesions) and fine-needle aspirate samples (suspected and early stage disease), and provides same day results (time-to-positive result <30 minutes) with simple interpretation. Of the outstanding requirements for further development, the incorporation of an internal control through multiplexing of assays as described above could be readily achieved. Numerous LAMP assays targeting other pathogens are already available in temperature-stable all-in-one dry reagents, removing the need for cold-chain transport (14, 63, 64). Application of existing lyophilization techniques to consolidate the IS*2404* P-LAMP assay into all-in-one dry reagents would further simplify the existing one-tube reaction.

The SARS-CoV-2 pandemic accelerated the development and distribution of multiple point-of-care diagnostic tests, including LAMP platforms suitable for use by non-skilled operators, with minimal training requirements (65). Advances in microfluidics have also sought to considerably simplify operator intervention through development of lab-on-chip technologies (66–68). Examples include a self-powered digital LAMP microfluidic chip for the detection of Zika virus (67), and a LAMP chip for in-field detection of tomato pathogens (68). Pairing of a multiplexed IS*2404* P-LAMP with such technology could reduce costs through miniaturization that requires less reagents and large-scale production for widespread use.

The implementation of multiplexed probe-based LAMP assays for the detection of *Treponema pallidum* (causative agent) and *Haemophilus ducreyi* (most common differential) to support the elimination of the neglected tropical disease, yaws, is currently undergoing feasibility trials across three African countries (69, 70). With development as discussed above, an improved iteration of the IS*2404* P-LAMP could potentially be a suitable diagnostic test to similarly guide efforts to eliminate BU.

## SUPPLEMENTAL MATERIAL

Supplemental material aem.00270-25-s0001.pdf Tables S1 to S4; Fig. S1 to S3.

ASM does not own the copyrights to Supplemental Material that may be linked to, or accessed through, an article. The authors have granted ASM a non-exclusive, world-wide license to publish the Supplemental Material files. Please contact the corresponding author directly for reuse.
