# Supplementary material for: Detection of Mycobacterium ulcerans with IS2404 loop-mediated isothermal amplification and a fluorescent reporter probe
Source: Appl Environ Microbiol. 2025 Apr 16;91(5):e00270-25. doi: 10.1128/aem.00270-25 (PMC12093978; doi:10.1128/aem.00270-25)
Supplement: Supplemental material — Tables S1 to S4; Fig. S1 to S3. [file aem.00270-25-s0001.pdf]

SUPPLEMENTARY MATERIAL FOR:

A probe-based loop-mediated isothermal amplification assay targeting IS*2404* for detection of *Mycobacterium ulcerans*.

Jean Y. H. Lee[1,2], Jessica L. Porter[1], Maria Globan[3], Caroline J. Lavender[3], Yinhua Zhang[4], Nathan A. Tanner[4], Emma C. Hobbs[5,6], Andrew H. Buultjens[1], and Timothy P. Stinear[1,3,*]

1. Department of Microbiology and Immunology, The University of Melbourne at the Doherty Institute for Infection and Immunity, Victoria, Australia.
2. Department of Infectious Diseases, Monash Health, Clayton, Victoria, Australia.
3. WHO Collaborating Centre for *Mycobacterium ulcerans*, Mycobacterium Reference Laboratory, Victorian Infectious Diseases Reference Laboratory, Doherty Institute, Melbourne, Victoria, Australia.
4. Applied Molecular Biology Research Division, New England Biolabs, Ipswich, Massachusetts, United States of America.
5. Melbourne Veterinary School, Department of Veterinary Biosciences, Faculty of Science, University of Melbourne, Werribee, Victoria, Australia.
6. Department of Infectious Diseases, University of Melbourne, The University of Melbourne at the Doherty Institute for Infection and Immunity, Victoria, Australia.

* Corresponding author: tstinear@unimelb.edu.au

Table of Contents

Supplementary Table 1. Bacterial strains used in this study. 3

Supplementary Figure 1. Binding sites of the IS*2404* TaqMan quantitative PCR (qPCR) and IS*2404* LAMP plus locked nucleic acid (LNA) probe (P-LAMP) primers and probes. 4

Supplementary Figure 2. Limit-of-detection (LoD) testing comparing the (**A**) IS*2404* probe LAMP (P-LAMP) and (**B**) IS*2404* quantitative PCR (qPCR) for the detection of *M. ulcerans* genomic DNA. 5

Supplementary Table 2. Collection site and IS*2404* qPCR cycle threshold (Ct) and P-LAMP time-to-positive (Tp) values for possum excreta surveillance samples. 6

Supplementary Table 3. IS*2404* qPCR cycle threshold (Ct) and P-LAMP time-to-positive (Tp) values for *M. ulcerans* DNA extracted from swabs sampled from Australian possums. 7

Supplementary Table 4. IS*2404* qPCR cycle threshold (Ct) and P-LAMP time-to-positive (Tp) values for *M. ulcerans* DNA extracted from human clinical swabs. 8

Supplementary Figure 3. Mic PCR consumables. 9

References 10

Supplementary Table 1. Bacterial strains used in this study.

Organism (strain)	Strain reference	Sequencing Reference
Mycobacterium ulcerans (JKD8049, clinical, Australia)	(1)	GCA_020616615.1
Mycobacterium ulcerans (Agy99, clinical, Ghana)	(2)	(2)
Mycobacterium ulcerans (ITM 06-3844, fish, Belgium)	(3)	(4)
Mycobacterium ulcerans (JKD8043, clinical, Suriname)	(5)	Not published
Mycobacterium ulcerans (Mu_L15, fish, USA)	(6)	(7)
Mycobacterium ulcerans (NM20/02, clinical, Ghana)	(8)	(4)
Mycobacterium abscessus (TPS8830)	Not published	Not published
Mycobacterium bovis (Danish 1331)	(9)	(10)
Mycobacterium chimera (DMG160013)	(11)	(11)
Mycobacterium fortuitum[#]	(12)	Not published
Mycobacterium marinum ("M" strain)	(13)	(13)
Mycobacterium smegmatis (MC2155)	(14)	(15)
Mycobacterium spongiae (FSD4b-SM)	(16)	(16)
Mycobacterium terrae (NCTC 10856/ ATCC 15981)	(17)	GCA_900187145.1
Mycobacterium virginiense (TPS8833)	Not published	Not published
Dendrosporobacter quercicolus (DSM 1736)	(18, 19)	Not published
Enterococcus faecium (Ef_AUS0233)	(20)	(20)
Escherichia coli (Ec_SeRP62aI)	(21)	(21)
Klebsiella pneumoniae (BPH05002)	Not published	Not published
Listeria monocytogenes (EGDe)	(22)	(23)
Nocardia brasiliensis (AUSMDU00075719)	Not published	Not published
Nocardia testacea (AUSMDU00041268)	Not published	Not published
Rouxiella chamberiensis (DSM 28324)	(24)	(25)
Staphylococcus aureus (JE2)	(26)	(27)
Staphylococcus aureus (MW2)	(28)	(29)
Staphylococcus epidermidis (BPH0662)	(30)	(30)
Staphylococcus epidermidis (BPH0736)	(31)	(31)
Streptomyces cacaoi (AUSMDU00077794)	Not published	Not published
Streptomyces spp. (AUSMDU00077800)	Not published	Not published

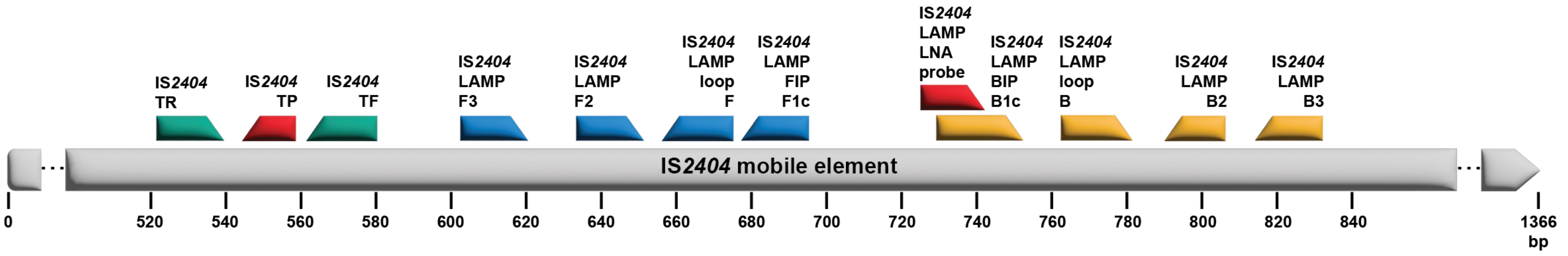

Supplementary Figure 1. Binding sites of the IS*2404* TaqMan quantitative PCR (qPCR) and IS*2404* LAMP plus locked nucleic acid (LNA) probe (P-LAMP) primers and probes. Note that primers have been named according to the publications in which they were originally described, in which IS*2404* TaqMan qPCR forward and reverse primers were named their reverse (32). The forwards and backward primers for the IS*2404* P-LAMP were also named the reverse of convention (33).

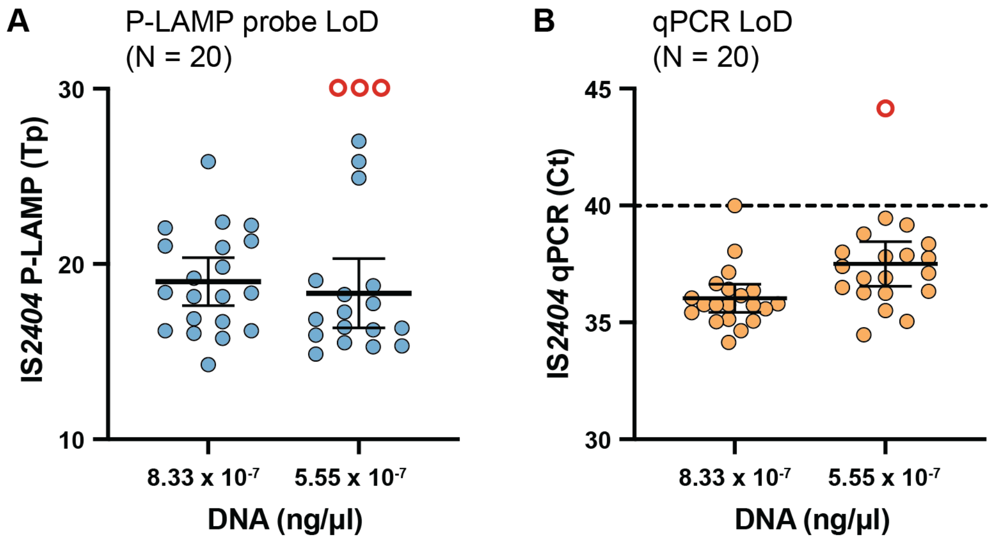

Supplementary Figure 2. Limit-of-detection (LoD) testing comparing the (A) IS*2404* probe LAMP (P-LAMP) and (B) IS*2404* quantitative PCR (qPCR) for the detection of *M. ulcerans* genomic DNA. Twenty replicates of each dilution were tested by each assay. Open red circles represent replicates that were not detected. Bold horizontal bars show the mean, error bars the 95% confidence interval. Horizontal dotted line indicates the cycle threshold (Ct) ≤40 cut-off for the IS*2404* qPCR, above which samples are considered as negative.

Supplementary Table 2. Collection site and IS*2404* qPCR cycle threshold (Ct) and P-LAMP time-to-positive (Tp) values for possum excreta surveillance samples.

Sample number	Collection site	IS*2404* qPCR (Ct)	IS*2404* P-LAMP (Tp)	Sample number	Collection site	IS*2404* qPCR (Ct)	IS*2404* P-LAMP (Tp)
1	RES19-02287	23.37	19.45	40	RES21-03709-T	32.78	19.15
2	RES19-02289	32.53	19.47	41	RES21-03709-U	33.76	21.36
3	RES19-02292	30.60	17.59	42	RES21-03709-V	ND	ND
4	RES21-00902	24.71	13.18	43	RES21-03709-W	32.33	18.65
5	RES21-00921	17.79	11.65	44	RES21-03709-X	32.10	21.14
6	RES21-00930	36.30	21.83	45	RES21-03709-Y	32.60	17.95
7	RES21-00993	34.43	17.00	46	RES21-03841	22.76	11.65
8	RES21-00999	19.56	17.37	47	RES21-03885	28.34	12.74
9	RES21-01067-A	22.41	15.47	48	RES21-03895	31.16	16.73
10	RES21-01067-B	27.64	12.60	49	RES22-03855-A	ND	ND
11	RES21-01079	23.55	12.33	50	RES22-03855-B	ND	ND
12	RES21-01131-A	28.78	13.37	51	RES22-03855-C	ND	ND
13	RES21-01131-B	30.55	16.92	52	RES22-03855-D	ND	ND
14	RES21-02262	ND	ND	53	RES22-03856-A	ND	ND
15	RES21-02291	31.14	19.68	54	RES22-03856-B	ND	ND
16	RES21-02636	34.22	16.11	55	RES22-03856-C	ND	ND
17	RES21-02644	37.10	19.90	56	RES22-03856-D	ND	ND
18	RES21-02662#	38.11	24.80	57	RES22-03856-E	ND	ND
19	RES21-03270	35.03	19.47	58	RES22-03856-E	ND	ND
20	RES21-03330	35.62	21.48	59	RES22-03856-F	ND	ND
21	RES21-03709-A	32.97	17.14	60	RES22-03856-G	ND	ND
22	RES21-03709-B	33.67	24.62	61	RES22-03856-H	ND	ND
23	RES21-03709-C	39.89	ND	62	RES22-03856-I	ND	ND
24	RES21-03709-D	28.17	16.58	63	RES22-03856-J	ND	ND
25	RES21-03709-E	32.41	18.68	64	RES22-03856-K	ND	ND
26	RES21-03709-F	33.39	18.95	65	RES22-03856-L	ND	ND
27	RES21-03709-G	32.92	21.43	66	RES22-03856-M	ND	ND
28	RES21-03709-H	32.71	17.41	67	RES22-03856-N	ND	ND
29	RES21-03709-I	32.76	20.44	68	RES22-03856-O	ND	ND
30	RES21-03709-J	38.37	ND	69	RES22-03856-P	ND	ND
31	RES21-03709-K	31.14	19.80	70	RES22-03856-Q	ND	ND
32	RES21-03709-L	33.12	23.30	71	RES22-03856-R	ND	ND
33	RES21-03709-M	34.96	18.40	72	RES22-03856-S	ND	ND
34	RES21-03709-N	32.41	21.37	73	RES22-03856-T	ND	ND
35	RES21-03709-O	31.42	20.13	74	RES22-03856-U	ND	ND
36	RES21-03709-P	32.75	24.50	75	RES22-03856-V	ND	ND
37	RES21-03709-Q	32.76	23.30	76	RES22-03856-W	ND	ND
38	RES21-03709-R	33.04	16.93	77	RES22-03856-X	ND	ND
39	RES21-03709-S	33.07	19.18	78	RES22-03856-Y	ND	ND

#SPRI-bead based DNA extraction tested instead of PowerSoil Pro kit extraction.

Supplementary Table 3. IS*2404* qPCR cycle threshold (Ct) and P-LAMP time-to-positive (Tp) values for *M. ulcerans* DNA extracted from swabs sampled from Australian possums.

Possum number (ID)	Swabbed site	IS*2404* qPCR (Ct)	IS*2404* P-LAMP (Tp)
1 (W379-23)	Multiple ulcers	ND	ND
2 (W381-23)	Multiple ulcers	ND	ND
3 (W386-23)	Multiple ulcers	ND	ND
4 (W387-23)	Multiple ulcers	ND	ND
5 (W380-23)	Multiple ulcers	19.81	10.33
6 (6/22)	Oral cavity	34.81	18.04
	Cloaca	27.85	13.39
7 (13/22)	Oral cavity	36.02	19.02
	Cloaca	ND	ND
8 (14/22)	Oral cavity	25.04	13.50
	Cloaca	32.58	13.50
	Pouch	33.02	16.06
9 (15/22)	Oral cavity	ND	ND
10 (20/23)	Oral cavity	32.98	19.53
	Cloaca	32.98	19.39
	Ulcer (L hind paw)	19.30	10.34

Ct: cycle threshold, ND: not detected, L: left.

Supplementary Table 4. IS*2404* qPCR cycle threshold (Ct) and P-LAMP time-to-positive (Tp) values for *M. ulcerans* DNA extracted from human clinical swabs

Sample	IS*2404* qPCR (Ct)	IS*2404* P-LAMP (Tp)	Sample	IS*2404* qPCR (Ct)	IS*2404* P-LAMP (Tp)	Sample	IS*2404* qPCR (Ct)	IS*2404* P-LAMP (Tp)
1	ND	ND	51	27.09	17.10	101	ND	ND
2	ND	ND	52	27.36	12.12	102	ND	ND
3	ND	ND	53	18.06	7.71			
4	ND	ND	54	ND	ND			
5	ND	ND	55	31.66	19.53			
6	ND	ND	56	32.21	14.28			
7	ND	ND	57	ND	ND			
8	ND	ND	58	26.48	14.64			
9	ND	ND	59	20.01	9.67			
10	ND	ND	60	18.57	10.88			
11	ND	ND	61	ND	ND			
12	ND	ND	62	ND	ND			
13	ND	ND	63	ND	ND			
14	ND	ND	64	ND	ND			
15	ND	ND	65	ND	ND			
16	ND	ND	66	ND	ND			
17	ND	ND	67	ND	ND			
18	ND	ND	68	ND	ND			
19	ND	ND	69	ND	ND			
20	ND	ND	70	ND	ND			
21	ND	ND	71	ND	ND			
22	ND	ND	72	ND	ND			
23	ND	ND	73	ND	ND			
24	ND	ND	74	ND	ND			
25	ND	ND	75	ND	ND			
26	ND	ND	76	ND	ND			
27	ND	ND	77	ND	ND			
28	ND	ND	78	ND	ND			
29	ND	ND	79	ND	ND			
30	ND	ND	80	ND	ND			
31	36.42	21.96	81	ND	ND			
32	34.82	19.01	82	ND	ND			
33	31.70	18.05	83	ND	ND			
34	23.57	10.57	84	ND	ND			
35	38.45	20.84	85	ND	ND			
36	25.16	11.04	86	ND	ND			
37	26.55	13.84	87	ND	ND			
38	ND	ND	88	ND	ND			
39	28.25	16.76	89	ND	ND			
40	26.32	16.43	90	ND	ND			
41	ND	ND	91	ND	ND			
42	ND	ND	92	ND	ND			
43	24.66	10.27	93	ND	ND			
44	38.57	23.93	94	ND	ND			
45	22.53	10.63	95	ND	ND			
46	32.02	27.89	96	ND	ND			
47	28.69	14.33	97	ND	ND			
48	34.88	18.12	98	ND	ND			
49	21.77	9.77	99	ND	ND			
50	31.63	15.36	100	ND	ND			

IS*2404*: insertion sequence *2404*, qPCR: quantitative polymerase chain reaction, LAMP: loop-mediated isothermal amplification.

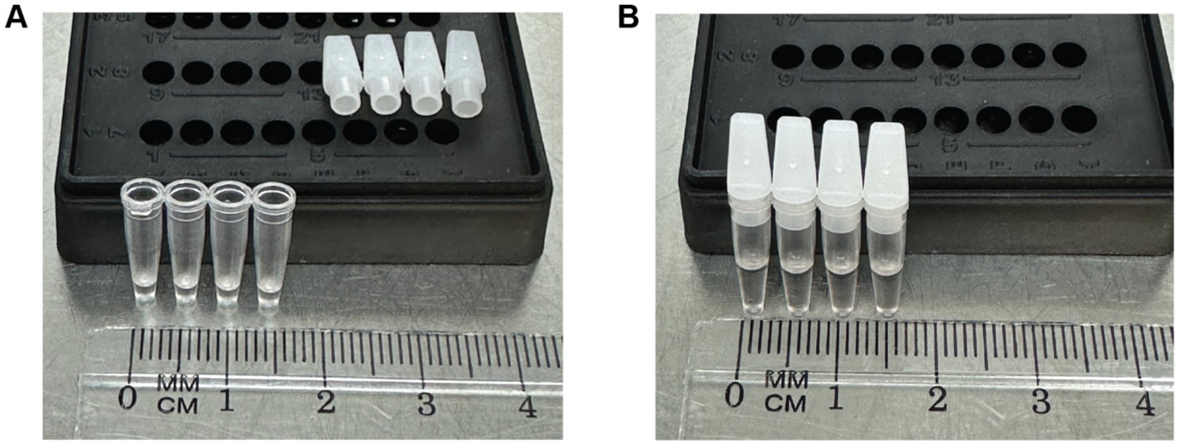

Supplementary Figure 3. Mic PCR consumables. (ruler for scale).

References

1. Doig KD, Holt KE, Fyfe JA, Lavender CJ, Eddyani M, Portaels F, Yeboah-Manu D, Pluschke G, Seemann T, Stinear TP. 2012. On the origin of *Mycobacterium ulcerans*, the causative agent of Buruli ulcer. BMC Genomics 13:258.
2. Stinear TP, Seemann T, Pidot S, Frigui W, Reysset G, Garnier T, Meurice G, Simon D, Bouchier C, Ma L, Tichit M, Porter JL, Ryan J, Johnson PD, Davies JK, Jenkin GA, Small PL, Jones LM, Tekaia F, Laval F, Daffe M, Parkhill J, Cole ST. 2007. Reductive evolution and niche adaptation inferred from the genome of *Mycobacterium ulcerans*, the causative agent of Buruli ulcer. Genome Res 17:192-200.
3. Stragier P, Hermans K, Stinear T, Portaels F. 2008. First report of a mycolactone-producing *Mycobacterium* infection in fish agriculture in Belgium. FEMS Microbiol Lett 286:93-5.
4. Muhi S, Buultjens AH, Porter JL, Marshall JL, Doerflinger M, Pidot SJ, O'Brien DP, Johnson PDR, Lavender CJ, Globan M, McCarthy J, Osowicki J, Stinear TP. 2024. *Mycobacterium ulcerans* challenge strain selection for a Buruli ulcer controlled human infection model. PLoS Negl Trop Dis 18:e0011979.
5. Faber WR, de Jong B, de Vries HJ, Zeegelaar JE, Portaels F. 2015. Buruli ulcer in traveler from Suriname, South America, to the Netherlands. Emerg Infect Dis 21:497-9.
6. Rhodes MW, Kator H, McNabb A, Deshayes C, Reyrat JM, Brown-Elliott BA, Wallace R, Trott KA, Parker JM, Lifland B, Osterhout G, Kaattari I, Reece K, Vogelbein W, Ottinger CA. 2005. *Mycobacterium pseudoshottsii* sp. nov., a slowly growing chromogenic species isolated from Chesapeake Bay striped bass (*Morone saxatilis*). Int J Syst Evol Microbiol 55:1139-1147.
7. Pidot SJ, Asiedu K, Kaser M, Fyfe JA, Stinear TP. 2010. *Mycobacterium ulcerans* and other mycolactone-producing mycobacteria should be considered a single species. PLoS Negl Trop Dis 4:e663.
8. Hilty M, Yeboah-Manu D, Boakye D, Mensah-Quainoo E, Rondini S, Schelling E, Ofori-Adjei D, Portaels F, Zinsstag J, Pluschke G. 2006. Genetic diversity in *Mycobacterium ulceran*s isolates from Ghana revealed by a newly identified locus containing a variable number of tandem repeats. J Bacteriol 188:1462-5.
9. Lugosi L. 1992. Theoretical and methodological aspects of BCG vaccine from the discovery of Calmette and Guerin to molecular biology. A review. Tuber Lung Dis 73:252-61.
10. Borgers K, Ou JY, Zheng PX, Tiels P, Van Hecke A, Plets E, Michielsen G, Festjens N, Callewaert N, Lin YC. 2019. Reference genome and comparative genome analysis for the WHO reference strain for *Mycobacterium bovis* BCG Danish, the present tuberculosis vaccine. BMC Genomics 20:561.
11. Williamson D, Howden B, Stinear T. 2017. *Mycobacterium chimaera* spread from heating and cooling units in heart surgery. N Engl J Med 376:600-602.
12. Stinear T, Ross BC, Davies JK, Marino L, Robins-Browne RM, Oppedisano F, Sievers A, Johnson PD. 1999. Identification and characterization of IS*2404* and IS*2606:* two distinct repeated sequences for detection of *Mycobacterium ulcerans* by PCR. J Clin Microbiol 37:1018-23.
13. Stinear TP, Seemann T, Harrison PF, Jenkin GA, Davies JK, Johnson PD, Abdellah Z, Arrowsmith C, Chillingworth T, Churcher C, Clarke K, Cronin A, Davis P, Goodhead I, Holroyd N, Jagels K, Lord A, Moule S, Mungall K, Norbertczak H, Quail MA, Rabbinowitsch E, Walker D, White B, Whitehead S, Small PL, Brosch R, Ramakrishnan L, Fischbach MA, Parkhill J, Cole ST. 2008. Insights from the complete

genome sequence of *Mycobacterium marinum* on the evolution of *Mycobacterium tuberculosis*. Genome Res 18:729-41.
14. Snapper SB, Melton RE, Mustafa S, Kieser T, Jacobs WR, Jr. 1990. Isolation and characterization of efficient plasmid transformation mutants of *Mycobacterium smegmatis*. Mol Microbiol 4:1911-9.
15. Mohan A, Padiadpu J, Baloni P, Chandra N. 2015. Complete genome sequences of a *Mycobacterium smegmatis* laboratory strain (MC2 155) and isoniazid-resistant (4XR1/R2) mutant strains. Genome Announc 3:e01520-14.
16. Pidot SJ, Klatt S, Ates LS, Frigui W, Sayes F, Majlessi L, Izumi H, Monk IR, Porter JL, Bennett-Wood V, Seemann T, Otter A, Taiaroa G, Cook GM, West N, Tobias NJ, Fuerst JA, Stutz MD, Pellegrini M, McConville M, Brosch R, Stinear TP. 2024. Marine sponge microbe provides insights into evolution and virulence of the tubercle bacillus. PLoS Pathog 20:e1012440.
17. Wayne LG. 1966. Classification and identification of *Mycobacteria*. 3. Species within group 3. Am Rev Respir Dis 93:919-28.
18. Stankewich JP, Cosenza BJ, Shigo AL. 1971. *Clostridium quercicolum* sp.n., isolated from discolored tissues in living oak trees. Antonie Van Leeuwenhoek 37:299-302.
19. Strompl C, Tindall BJ, Lunsdorf H, Wong TY, Moore ER, Hippe H. 2000. Reclassification of *Clostridium quercicolum* as *Dendrosporobacter quercicolus* gen. nov., comb. nov. Int J Syst Evol Microbiol 50 Pt 1:101-106.
20. Buultjens AH, Lam MM, Ballard S, Monk IR, Mahony AA, Grabsch EA, Grayson ML, Pang S, Coombs GW, Robinson JO, Seemann T, Johnson PD, Howden BP, Stinear TP. 2017. Evolutionary origins of the emergent ST796 clone of vancomycin resistant *Enterococcus faecium*. PeerJ 5:e2916.
21. Lee JYH, Carter GP, Pidot SJ, Guerillot R, Seemann T, Goncalves da Silva A, Foster TJ, Howden BP, Stinear TP, Monk IR. 2019. Mining the methylome reveals extensive diversity in *Staphylococcus epidermidis* restriction modification. mBio 10:e02451-19.
22. Murray EGD, Webb RA, Swann MBR. 1926. A disease of rabbits characterised by a large mononuclear leucocytosis, caused by a hitherto undescribed bacillus *Bacterium monocytogenes* (n.sp.). The Journal of Pathology and Bacteriology 29:407-439.
23. Glaser P, Frangeul L, Buchrieser C, Rusniok C, Amend A, Baquero F, Berche P, Bloecker H, Brandt P, Chakraborty T, Charbit A, Chetouani F, Couve E, de Daruvar A, Dehoux P, Domann E, Dominguez-Bernal G, Duchaud E, Durant L, Dussurget O, Entian KD, Fsihi H, Garcia-del Portillo F, Garrido P, Gautier L, Goebel W, Gomez-Lopez N, Hain T, Hauf J, Jackson D, Jones LM, Kaerst U, Kreft J, Kuhn M, Kunst F, Kurapkat G, Madueno E, Maitournam A, Vicente JM, Ng E, Nedjari H, Nordsiek G, Novella S, de Pablos B, Perez-Diaz JC, Purcell R, Remmel B, Rose M, Schlueter T, Simoes N, et al. 2001. Comparative genomics of *Listeria* species. Science 294:849-52.
24. Le Fleche-Mateos A, Levast M, Lomprez F, Arnoux Y, Andonian C, Perraud M, Vincent V, Ar Gouilh M, Thiberge JM, Vandenbogaert M, Diancourt L, Caro V, Burguiere AM, Manuguerra JC. 2015. *Rouxiella chamberiensis* gen. nov., sp. nov., a member of the family Enterobacteriaceae isolated from parenteral nutrition bags. Int J Syst Evol Microbiol 65:1812-1818.
25. Paul S, Anderson PJ, Maynard GJ, Dyall-Smith M, Kudinha T. 2023. Complete genome sequences of the type strains *Rouxiella badensis* DSM 100043 and *R. chamberiensis* DSM 28324, resolved using nanopore long-read sequencing. Microbiol Resour Announc 12:e0015623.
26. Fey PD, Endres JL, Yajjala VK, Widhelm TJ, Boissy RJ, Bose JL, Bayles KW. 2013. A genetic resource for rapid and comprehensive phenotype screening of nonessential *Staphylococcus aureus* genes. mBio 4:e00537-12.

27. Bernardy EE, Petit RA, 3rd, Moller AG, Blumenthal JA, McAdam AJ, Priebe GP, Chande AT, Rishishwar L, Jordan IK, Read TD, Goldberg JB. 2019. Whole-genome sequences of *Staphylococcus aureus* isolates from cystic fibrosis lung infections. Microbiol Resour Announc 8(3):e01564-18.
28. Centers for Disease C, Prevention. 1999. Four pediatric deaths from community-acquired methicillin-resistant *Staphylococcus aureus* - Minnesota and North Dakota, 1997-1999. MMWR Morb Mortal Wkly Rep 48:707-10.
29. Baba T, Takeuchi F, Kuroda M, Yuzawa H, Aoki K, Oguchi A, Nagai Y, Iwama N, Asano K, Naimi T, Kuroda H, Cui L, Yamamoto K, Hiramatsu K. 2002. Genome and virulence determinants of high virulence community-acquired MRSA. Lancet 359:1819-27.
30. Lee JYH, Monk IR, Pidot SJ, Singh S, Chua KYL, Seemann T, Stinear TP, Howden BP. 2016. Functional analysis of the first complete genome sequence of a multidrug resistant sequence type 2 *Staphylococcus epidermidis*. Microb Genom 2:e000077.
31. Lee JYH, Monk IR, Goncalves da Silva A, Seemann T, Chua KYL, Kearns A, Hill R, Woodford N, Bartels MD, Strommenger B, Laurent F, Dodemont M, Deplano A, Patel R, Larsen AR, Korman TM, Stinear TP, Howden BP. 2018. Global spread of three multidrug-resistant lineages of *Staphylococcus epidermidis*. Nat Microbiol 3:1175-1185.
32. Fyfe JA, Lavender CJ, Johnson PD, Globan M, Sievers A, Azuolas J, Stinear TP. 2007. Development and application of two multiplex real-time PCR assays for the detection of *Mycobacterium ulcerans* in clinical and environmental samples. Appl Environ Microbiol 73:4733-40.
33. Buultjens AH, Vandelannoote K, Sharkey LK, Howden BP, Monk IR, Lee JYH, Stinear TP. 2021. Low-cost, open-source device for high-performance fluorescence detection of isothermal nucleic acid amplification reactions. ACS Biomater Sci Eng 7:4982-4990.
